# The influence of real-time feedback on the quality of resuscitation: A prospective study comparing bystanders, paramedic course participants, and emergency physician trainees

**DOI:** 10.3205/zma001790

**Published:** 2025-11-17

**Authors:** Stella-Karolin Krispin, Anja Haase-Fielitz, Grit Spalding, Jana Steigerwald, Lars Trenkmann

**Affiliations:** 1 Universitätsklinikum Ruppin- Brandenburg, Universitätsklinikum der Medizinischen Hochschule Brandenburg Theodor Fontane, Klinik für Anästhesie und Intensivmedizin, Neuruppin, Germany; 2 Immanuel Klinikum Bernau Herzzentrum Brandenburg, bteilung für Kardiologie, Universitätsklinikum der Medizinischen Hochschule Brandenburg Theodor Fontane, Bernau, Germany; 3 Gemeinsame Fakultät der Universität Potsdam, der Brandenburgischen Medizinischen Hochschule Theodor Fontane und der Brandenburgischen Technischen Universität Cottbus-Senftenberg, Fakultät für Gesundheitswissenschaften (FGW), Cottbus, Germany; 4 Otto-von-Guericke-Universität Magdeburg, Institut für Sozialmedizin und Gesundheitssystemforschung, Magdeburg, Germany; 5 Immanuel Klinikum Bernau Herzzentrum Brandenburg, Universitätsklinikum der Medizinischen Hochschule Brandenburg Theodor Fontane, Zentrale Notaufnahme, Bernau, Germany; 6 Klein-Winternheim, Germany; 7 Charité – Universitätsmedizin Berlin, Klinik für Nephrologie und internistische Intensivmedizin, Berlin, Germany; 8 Notfallmedizinische Aus- und Weiterbildung Berlin (NAW Berlin), Staatlich anerkannte Rettungsdienstschule, Berlin, Germany

**Keywords:** resuscitation, chest compression, feedback, bystander

## Abstract

**Objective:**

The aim of this study was to analyze the potential benefits of real-time feedback in resuscitation training for participants in the prehospital emergency chain and to compare differences in the quality of chest compressions (CC) with and without feedback.

**Methods:**

The primary endpoint was to analyze the proportion of CC achieving the recommended depth (5-6cm) and frequency (100-120/min) during two minutes of CC. This prospective cohort study compares bystanders (N=75), paramedic trainees (N=75), and emergency physician trainees (N=75) with and without the feedback system of the Zoll X-Series^®^.

**Results:**

Without feedback, paramedics (P) achieved the target compression frequency in 82.7%, bystanders (B) in 49.8%, and emergency physician trainees (EP) in 75% (P vs. B, p<0.001; EP vs. P, p=0.759; EP vs. B, p=0.217). There were no significant differences in target compression depth without feedback.

With feedback, P achieved the compression frequency in 90.7%, B in 72.8%, and EP in 91.4% (P vs. B, p<0.001; EP vs. P, p=0.425; EP vs. B, p<0.001).

With feedback, P achieved the compression depth in 56.9%, B in 47.3%, and EP in 75.1% (P vs. B, p=0.759; EP vs. P, p=0.217; EP vs. B, p=0.002).

**Conclusion:**

The results underscore the importance of real-time feedback in emergency medical training, especially for B. All cohorts showed significant improvement, indicating that feedback enhances CC and promotes skill development. Given the importance of high-quality CC, their early optimization in training is essential. This highlights the need for standardized training concepts, including timing recommendations for feedback systems. Future studies should consider real-life prehospital conditions and investigate chest compression to validate transferability to real-life scenarios.

## Zusammenfassung

**Zielsetzung:**

Ziel der Studie war es, den möglichen Nutzen von Echtzeitfeedback in der Reanimationsausbildung von Beteiligten der präklinischen Rettungskette zu analysieren und Unterschiede in der Qualität der Herzdruckmassage (HDM) ohne und mit Feedback zu vergleichen.

**Methoden:**

Primärer Endpunkt war der Anteil der Thoraxkompressionstiefe (5-6cm) und – frequenz (100-120/min) während zweiminütiger HDM zu analysieren. Diese prospektive Kohortenstudie vergleicht Ersthelfende (N=75), angehenden Rettungssanitäter:innen (N=75) und Notärzt:innen (N=75) ohne und mit Feedbacksystem der Zoll X-Series^®^.

**Ergebnisse:**

Ohne Feedback erreichten RS die Zieldruckfrequenz in 82,7%, EH in 49,8% und NÄ in 75% (RS vs. EH p<0,001; NÄ vs. RS p=0,759; NÄ vs. EH p=0,217). Die Zieldrucktiefe ergab keine signifikanten Unterschiede ohne Feedback.

Mit Feedback erreichten RS die Druckfrequenz in 90,7%, EH in 72,8% und NÄ in 91,4% (RS vs. EH p<0,001; NÄ vs. RS p=0,425; NÄ vs. EH p<0,001).

Mit Feedback erreichten RS die Drucktiefe in 56,9%, EH in 47,3% und NÄ in 75,1% (RS vs. EH p=0,759; NÄ vs. RS p=0,217; NÄ vs. EH p=0,002).

**Schlussfolgerung:**

Die Ergebnisse belegen die Relevanz von Echtzeitfeedback in notfallmedizinischer Ausbildung, insbesondere für Ersthelfende. Alle Kohorten profitierten signifikant, sodass Feedback die HDM verbessern und außerdem den Kompetenzaufbau fördert. Angesichts der Bedeutung einer hochwertigen HDM ist deren frühzeitige Optimierung bereits im Training essenziell. Daraus ergibt sich der Bedarf, standardisierter Schulungskonzepte inklusive Empfehlungen zum Anwendungszeitpunkt von Feedbacksystemen. Künftige Studien sollten reale, präklinische Bedingungen einbeziehen, zusätzlich die Thoraxentlastung untersuchen, um die Übertragbarkeit auf den Einsatzalltag zu validieren.

## Introduction

As soon as a cardiac arrest occurs, quick action is essential to restore circulation and prevent neurological damage [1], [2]. In 2023, approximately 25.500 prehospital resuscitations were documented [3]. Around 10% of prehospital resuscitated patients were discharged from the hospital [3]. The first step in the chain of survival is to immediately call for emergency assistance while simultaneously beginning resuscitation [4], [5]. On average, emergency services arrived after six minutes and 48 seconds in 2023 [3]. Since this period without circulation is sufficient to cause irreversible brain damage, immediate chest compressions by bystanders are critical for survival. Therefore, layperson resuscitation plays a crucial role until professional rescuers take over the resuscitation. In addition to early defibrillation for shockable arrhythmias, chest compressions (CC) are the most important measure for the treatment of cardiac arrest. The German Resuscitation Council (GRC) guidelines recommend a two-minute rescuer rotation and define five parameters for high-quality CC [6]. These parameters include correct hand placement (lower third of the sternum), a compression depth of 5-6 cm, a compression rate of 100-120 compressions per minute, complete chest recoil, and compression pauses shorter than 10 seconds. It is known that CC are often performed inadequately [7]. Therefore, the ERC guidelines recommend regular resuscitation training and the use of real-time feedback [8]. Feedback systems evaluate the quality of CC in real time, compare it with the required parameters, and provide (audio-)visual feedback, allowing adjustments to the chest compressions if necessary [9], [10]. Early studies investigating the effectiveness of various feedback systems for lay rescuers and medical personnel found that CC were performed with better quality when aided by prompt feedback [10]. For example, lay rescuers in the study by Krasteva et al. showed a significant improvement in compression quality over a three-minute period [11]. Nursing students also benefited significantly from the use of real-time feedback, according to Smereka et al. [12]. Cheng et al. and Buléon et al. reported similar effects in experienced healthcare professionals [13], [14], [15]. A recent systematic review by Nicolau et al. confirms these findings: corrective feedback systems significantly improve compression depth and rate, regardless of the users' experience level [16]. The comparability of the studies published so far is limited, as they vary significantly in terms of settings, compression duration, and the composition of participants. Moreover, few of the existing studies differentiate between the specific professional groups within the prehospital emergency chain. The study by Hostler et al. demonstrates the benefits of feedback systems in prehospital settings but remains unspecific regarding the professional roles within the rescue team [17]. In contrast to the studies included in the review by Nicolau et al., this study addresses an underrepresented target group – prehospital personnel – under standardized simulation conditions. Specifically, lay rescuers and emergency medical services provide the initial care for patients in cardiac arrest, meaning the quality of the initial care significantly impacts their prognosis. At the time of the literature review, no studies were known that directly compared these groups involved in the chain of survival. Therefore, this study investigates whether there are differences in the quality of chest compressions between bystanders, paramedic trainees, and emergency physician trainees – and whether the integration of prompt feedback affects resuscitation quality in these three groups. The hypothesis was that lay rescuers using real-time feedback would achieve a comparable quality of CC to that of medical professionals without the use of feedback.

## Methods

### Study design and sample size

This study was designed as a prospective cohort study. The sample size calculation is based on the work of Buléon et al. [15]. With a significance level of α=0.05, an expected effect size of d=1.0, and an assumed difference of 21%, a minimum group size of n=20 was required to achieve a power of 0.80 [15]. To increase the statistical power, account for potential missing data, and allow for differentiated group comparisons, 75 participants were included in each cohort.

### Participants/study population

Recruitment and data collection took place at the state-recognized emergency medical service school "NAW Berlin". A total of 225 participants from the first aid courses (Bystanders), paramedic courses (Paramedics), and emergency physician courses (Emergency Physicians) voluntarily participated in the study outside of their regular class hours. Before the study began, the bystanders received theoretical knowledge on how to perform chest compressions (CC). The paramedic cohort had completed a four-week theoretical and practical course at the time of data collection and was about to begin hospital and emergency service internships. The emergency physician cohort consisted of licensed physicians attending an emergency physician course.

### Study setting and procedure

The resuscitation simulator AmbuMan^®^ was equipped with the Zoll X-Series^®^ feedback system, thus standardizing the hand placement. Initially, participants performed two minutes of CC without visibility of the prompt feedback. After a two-minute break, another two minutes of CC were performed, this time with real-time feedback. All resuscitations were conducted under standardized conditions to minimize external influencing factors.

### Data collection

Data collection took place from October 2020 to January 2021. The focus of the resuscitation analysis was on the evaluation of compression depth (CD) within the target range (TR) and compression rate (CR) within the target range (TR). The reference values are provided by the resuscitation guidelines [5]. The compression depth should be between 5-6 cm, and the compression rate should be between 100-120 compressions per minute [5]. “Above the target range” (aTR) referred to compressions with a depth >6 cm or a rate >120 compressions per minute. “Below the target range” (bTR) defined a compression depth of less than 5 cm or a rate of fewer than 100 compressions per minute. Full chest recoil was visually displayed by the feedback system (Zoll X-Series^®^) but was not captured as an exportable parameter by the evaluation software (RescueNet Code Review). Since no quantitatively analyzable data on chest recoil were available, the study focused on the objectively measurable, guideline-relevant parameters of compression depth and compression rate. The manufacturer specifies a measurement accuracy of ±0.6 cm for compression depth within the range of 1.9-7.6 cm. The measurement is based on the integration of an accelerometer, which uses filtering algorithms (e.g., Kalman filter) to compensate for disturbances. In a previous functional check, the compression depth and compression rate of the Zoll X-Series^®^ feedback system were tested. The display of compression depth was compared to the reference display of the simulator, while the compression rate was additionally validated with an external app for frequency measurement. In both cases, the measurements were consistent, suggesting reliable detection of the relevant parameters by the feedback system.

### Data analysis and statistics

The analysis of the results was performed using the “RescueNet Code Review” program, which compares compression depth and frequency with the predefined guideline parameters. The statistical analysis of the collected data was conducted using the statistical software “SPSS Version 28 (IBM)”. To describe the central tendency with skewed data distribution and ordinal data, the median with the 25.-75. percentile was used. The comparison of the three independent groups was conducted using the Mann-Whitney U test, as the data were not normally distributed. Categorical variables were compared using the chi-square test. The results are presented in tables and graphs as boxplots or as absolute numbers with percentage values. The reporting of this prospective cohort study follows the STROBE checklist (Strengthening the Reporting of Observational Studies in Epidemiology) [18].

### Ethics

The study was approved by the relevant ethics committee of the Brandenburg Medical School before the start of data collection (ethics approval E-01-20200403). All participants were informed in advance and provided written consent to participate in the study. The conduct of the study adhered to the ethical standards of the Declaration of Helsinki.

## Results

Data from a total of 225 participants, consisting of participants from a first aid course (N=75), a paramedic course (N=75), and an emergency physician course (N=75), were analyzed. The participants were comparable in terms of gender distribution, with approximately 40% female participants in each group (see table 1 (Tab. 1)); participants from the emergency physician course were older than those from the first aid and paramedic courses (see table 1 (Tab. 1)). The guidelines specify 5 parameters to be followed for CC [5]. The correct hand placement was ensured by the prior attachment of the feedback system on the simulator. The analysis focused on the two parameters of compression depth and compression rate.

### Quality of chest compression without feedback system

The target compression depth was achieved by the bystanders (B) with a median of 12.4%, by the paramedics (P) with 18.4%, and by the emergency physicians (EP) with 15.5% (B vs. P vs. EP: p=0.304). Above the target range for compression depth, B performed compressions with a median of 85% and EP with 16.1%, while P did not perform any compressions that were too shallow. No compressions below the target range for compression depth were performed by the B, while P performed compressions too shallow with a median of 62.7%, and EP with 2.4%.

In the analysis of compression frequency, bystanders reached the target range with a median of 49.8%. P performed compressions within the target range with a median of 82.7%, and EP with 75%. A significant difference was found between B vs. P, with p<0.001. Above the target range for compression frequency, B performed compressions with a median of 4.2%, P with 4.6%, and EP with 5.3%. Below the target range, B performed compressions with a median of 2.3%, P with 0.4%, and EP with 0.4%.

### Quality of chest compression with feedback system

While using the feedback system, B performed compressions within the target range for compression depth with a median of 47.3%, P with 56.9%, and EP with 75.1%. A statistically significant difference was found between B and EP (p=0.002).

Above the target range for compression depth, B performed compressions with a median of 15%, and EP with 4.7%, while P did not perform any compressions that were too deep. Below the target range for compression depth, B performed compressions with a median of 10.6%, P with 24.8%, and EP with 3.6%. In terms of compression frequency, B performed compressions within the target range with a median of 72.8%, P with 90.7%, and EP with 91.4%. The results between B vs. P and B vs. EP were statistically significantly different with p<0.001. Above the target range for compression frequency, B performed compressions with a median of 16.2%, P with 6.4%, and EP with 0.5%. Below the target range, B performed compressions with a median of 1.5%, P with 0.4%, and EP with 0.5%.

**Figure 1:**
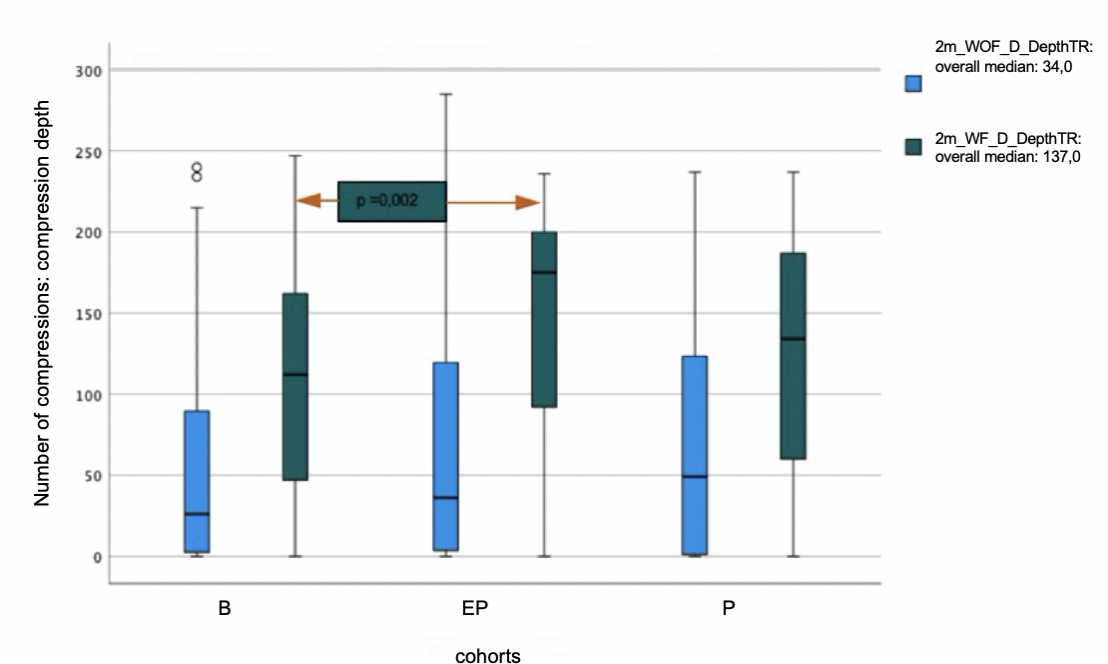
Comparison of the median compression depth within the target range (TR) with (WF) and without (WOF) feedback system between bystanders (B), emergency physician trainees (EP), and paramedic trainees (P)

**Figure 2:**
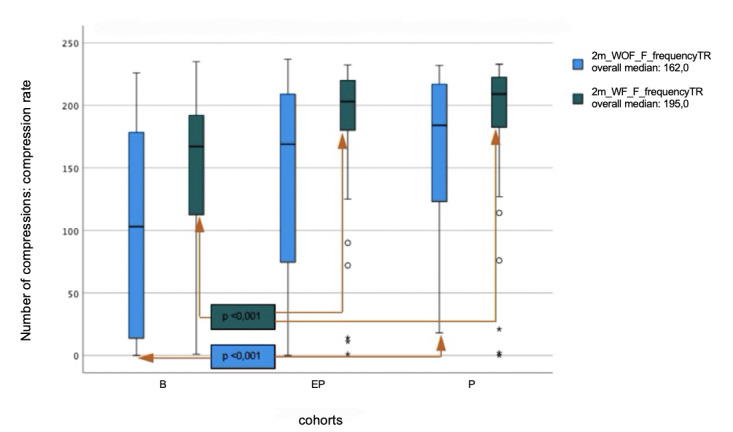
Comparison of the median compression rate within the target range (TR) with (W) and without (WOF) feedback system between bystanders (B), emergency physician trainees (EP), and paramedic trainees (P)

There was no significant difference between the P and EP cohorts in the analysis of either compression depth or compression frequency. Regarding the evaluated parameters, all cohorts were able to improve the quality of chest compressions with real-time feedback. In particular, the bystander cohort benefited from prompt feedback. The comparisons between the cohorts are shown in figures 1 (Fig. 1) and figure 2 (Fig. 2).

## Discussion

This study analyzes the quality of chest compressions among participants in the prehospital emergency chain, comparing bystanders (B), paramedic trainees (P), and emergency physician trainees (EP). All groups were able to significantly improve their CC performance with the help of real-time feedback. B reached a level of CC quality through feedback that was comparable to the CC quality of P without feedback.

Even without the feedback system, significant differences were observed: B achieved the target compression depth in a median of 12.4% of compressions, paramedics in 18.4%, and EP in 15.5%. In terms of compression frequency, B were significantly behind P at 82.7% and EP at 75%, with 49.8%. These results are in line with previous studies, such as those by Krasteva et al. and Cheng et al., which demonstrate the effectiveness of feedback systems in improving chest compression quality –both for laypersons and medical professionals [11], [13], [14]. The meta-analysis by Nicolau et al. demonstrates the effectiveness of feedback systems in improving compression depth and frequency, regardless of the experience level of the learners [16]. In contrast to the studies included in that analysis, the present investigation specifically focuses on prehospital personnel and is based on a uniformly structured simulation protocol. The duration of chest compressions was standardized to two minutes, in line with the recommendations of the current resuscitation guidelines [4], [5], [7], [19]. The study design is aligned with the practical requirements of resuscitation execution and enhances comparability with current guideline recommendations. At the same time, this methodological approach minimizes biases, such as those criticized in the meta-analysis by Nicolau et al., which arose from including studies with inconsistent study protocols and unclear definitions of feedback mechanisms. The guidelines recommend the use of feedback systems in the training of both laypersons and medical professionals, but lack specific instructions regarding the use of the devices [8]. Neither in the flowcharts of the training materials nor in the “step-by-step” instructions of the GRC guidelines is it specified when the feedback sensors should be applied [8], [20]. Furthermore, both first aid training and paramedic training in Germany are not standardized [21], [22]. Therefore, it is questionable to what extent the use of feedback systems is actually included in resuscitation training. Based on the presented results, we recommend the integration of real-time feedback into training materials and emergency medical education. Another possible explanation for the improvement in chest compression quality through real-time feedback lies in tactile learning. According to the review by Marchal-Crespo and Reinkensmeyer, tactile feedback can specifically enhance motor skills, especially when visual or auditory feedback alone is insufficient [23]. Applied to CC, real-time feedback could help participants develop a tactile sense for compression depth and rate, allowing them to immediately correct these parameters during performance. Without feedback, it may be difficult to correctly assess the required target values due to individual chest anatomy and possibly a lack of time perception in stressful situations. This assumption is supported by the study by Miotto et al., which shows that theoretical training alone is not sufficient to develop the psychomotor skills required for high-quality CC [24]. Practical exercises – and here, real-time feedback – allow participants to directly assess and adjust their technique. Furthermore, the feedback system may have enhanced participants' attention and motivation, as they could continuously monitor their performance. This provides a valuable learning mechanism, especially for inexperienced bystanders. The design of the study – first performing chest compressions (CC) without feedback, then with feedback – may have amplified this effect, as participants could specifically improve their initial assessments in the second round. In addition to the previously discussed aspects explaining the heterogeneous results between the cohorts, the different task distribution within the rescue team should also be considered. The crew resource management concept designates a leadership role in emergency situations [25]. The role of team leader during a resuscitation is often taken on by the emergency physicians [26]. This distribution of tasks suggests that paramedics are more likely to perform CC themselves, while EP, in addition to providing medical interventions, coordinate the team. EP have the highest level of emergency medical qualification among the responders due to their extensive training. In smaller teams, it may be necessary for them to perform chest compressions themselves after two minutes due to the required rescuer rotation. Therefore, the proper execution of chest compressions should be assumed by all participants in the chain of survival, starting with bystanders and extending to the rescue team. It is known that frequent resuscitation training improves compression quality [27]. 

Accordingly, the lack of training in the first aid area or during the incomplete P and EP training could have influenced the quality of chest compressions. Despite participants being in training, those with emergency medical background knowledge tended to perform better, emphasizing the relevance of feedback systems, especially for B.

It is particularly noteworthy that all cohorts, regardless of their prior experience, benefited from real-time feedback. This underscores its importance, particularly for the initial links in the chain of survival. This finding is consistent with the results of Nicolau et al., who reported that real-time feedback not only improves compression quality in the short term but also has a learning-enhancing effect on motor and tactile skills – particularly in compact, well-structured training sessions.

### Limitations

At the same time, some methodological limitations must be considered: The analysis focused on compression depth and frequency. Additionally, the measurement relied on accelerometers, which could be influenced by external factors. The use of a “within-subjects” design minimized individual differences but does not completely exclude a minor learning effect due to the fixed test order. The potential effect of different prior training of emergency physicians from various medical disciplines was not analyzed in the study due to the group size and should be investigated in future studies. Future studies should also consider chest recoil and examine the use of feedback systems under real-life, prehospital conditions to better assess the transferability to everyday practice.

## Conclusion

In summary, the results reinforce the relevance of real-time feedback in emergency medical training and support its integration into future training concepts.

## Authors’ ORCIDs

- Stella-Karolin Krispin: [0009-0001-7798-6571]
- Anja Haase-Fielitz: [0000-0001-6881-2249]
- Lars Trenkmann: [0009-0006-7770-7022]

## Data

Data for this article are available from Dryad Repository: [https://doi.org/10.5061/dryad.9w0vt4brg [28]

## Competing interests

The authors declare that they have no competing interests. 

For data collection, the resuscitation simulator AmbuMan^®^ was provided free of charge by the NAW Berlin Emergency Medical Service School. The company Zoll^®^ also provided the feedback system of the Zoll X-Series^®^ free of charge.

## Figures and Tables

**Table 1:**
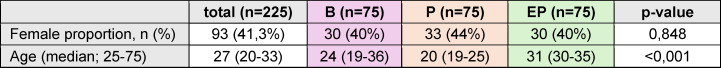
Characteristics of the study population
